# L-arginine availability and arginase activity: Characterization of amino acid permease 3 in *Leishmania amazonensis*

**DOI:** 10.1371/journal.pntd.0006025

**Published:** 2017-10-26

**Authors:** Juliana Ide Aoki, Sandra Marcia Muxel, Ricardo Andrade Zampieri, Stephanie Maia Acuña, Juliane Cristina Ribeiro Fernandes, Rubia Heloisa Vanderlinde, Maria Carmen Oliveira de Pinho Sales, Lucile Maria Floeter-Winter

**Affiliations:** Departamento de Fisiologia, Instituto de Biociências, Universidade de São Paulo, São Paulo, Brazil; McGill University, CANADA

## Abstract

**Background:**

*Leishmania* uses the amino acid L-arginine as a substrate for arginase, enzyme that produces urea and ornithine, last precursor of polyamine pathway. This pathway is used by the parasite to replicate and it is essential to establish the infection in the mammalian host. L-arginine is not synthesized by the parasite, so its uptake occurs through the amino acid permease 3 (AAP3). AAP3 is codified by two copies genes (*5*.*1* and *4*.*7* copies), organized in tandem in the parasite genome. One copy presents the expression regulated by L-arginine availability.

**Methodology/Principal findings:**

RNA-seq data revealed 14 amino acid transporters differentially expressed in the comparison of *La*-WT vs. *La*-arg^-^ promastigotes and axenic amastigotes. The *5*.*1* and *4*.*7 aap3* transcripts were down-regulated in *La*-WT promastigotes vs. axenic amastigotes, and in *La*-WT vs. *La*-arg^-^ promastigotes. In contrast, transcripts of other transporters were up-regulated in the same comparisons. The amount of *5*.*1* and *4*.*7 aap3* mRNA of intracellular amastigotes was also determined in sample preparations from macrophages, obtained from BALB/c and C57BL/6 mice and the human THP-1 lineage infected with *La*-WT or *La*-arg^-^, revealing that the genetic host background is also important. We also determined the *aap3* mRNA and AAP3 protein amounts of promastigotes and axenic amastigotes in different environmental growth conditions, varying pH, temperature and L-arginine availability. Interestingly, the increase of temperature increased the AAP3 level in plasma membrane and consequently the L-arginine uptake, independently of pH and L-arginine availability. In addition, we demonstrated that besides the plasma membrane localization, AAP3 was also localized in the glycosome of *L*. *amazonensis* promastigotes and axenic amastigotes.

**Conclusions/Significance:**

In this report, we described the differential transcriptional profiling of amino acids transporters from *La*-WT and *La*-arg^-^ promastigotes and axenic amastigotes. We also showed the increased AAP3 levels under amino acid starvation or its decrease in L-arginine supplementation. The differential AAP3 expression was determined in the differentiation of promastigotes to amastigotes conditions, as well as the detection of AAP3 in the plasma membrane reflecting in the L-arginine uptake. Our data suggest that depending on the amino acid pool and arginase activity, *Leishmania* senses and could use an alternative route for the amino acid transport in response to stress signaling.

## Author summary

*Leishmania* alternates its life cycle between the invertebrate host, in which the promastigote forms reside at pH 7.0 and approximately 25°C; and the mammalian host, in which the amastigote forms reside at pH 5.0 and approximately 37°C. These environmental changes submit the parasite to dynamic undergo modifications in morphology, metabolism, cellular signaling and gene expression regulation to allow for a rapid adaptation to the new environmental conditions. *Leishmania* is auxotrophic for many amino acids, such as L-arginine. The L-arginine availability is important for the uptake control by the parasite as well as the intracellular amino acids pool maintenance. *Leishmania* arginase uses L-arginine to produce urea and ornithine, being the last one a precursor of polyamine biosynthetic pathway, which is used by the parasite to replicate and to establish the infection. L-arginine uptake in *L*. *amazonensis* is performed through AAP3, which is encoded by two gene copies arranged *in tandem* on the genome (*5*.*1* and *4*.*7 aap3*). In this report, we characterized the AAP3 function and expression regulation in parasites maintained in different pH and temperature conditions simulating both insect and mammalian micro-environment. We submitted the parasites to amino acid starvation, simulating mid-gut starvation, a signal for promastigote metacyclogenesis. Our results demonstrated that the changes in temperature and pH, in addition with amino acid starvation or L-arginine supplementation might represent important signals for *aap3* expression regulation, mainly for the *5*.*1 aap3* copy. We also linked the regulation of *5*.*1 aap3* transcript to the genetic background from the host macrophage. In addition, we localized the AAP3 in the plasma membrane and in the glycosome of *L*. *amazonensis* promastigotes and axenic amastigotes, indicating that arginine uptake is directed to this organelle.

## Introduction

*Leishmania* is the causative agent of leishmaniasis, a complex disease characterized by cutaneous, mucocutaneous or visceral lesions [1–3]. It is currently endemic in 98 countries and territories around the world, with an annual incidence estimated at 1 million cases of cutaneous leishmaniasis and 300,000 cases of visceral leishmaniasis [4]. In its life cycle, the parasite alternates between the intestinal tract of the sand fly (promastigote form) and the phagolysosome compartment of mammalian host macrophages (amastigote form). These environmental changes submit the parasite to undergo extensive modifications and can be considered a trigger for the gene expression regulations that lead to adaptation to the new milieu.

*Leishmania* infection results in the specific activation of mammalian immune responses. Macrophages have a fundamental role in infection as the first line of defense. The production of nitric oxide (NO), a potent molecule effective against pathogens, is one of the key defense mechanisms of mammalian phagocytes [5]. NO is produced by nitric oxide synthase 2 (NOS2) using the amino acid L-arginine as substrate. Several studies have demonstrated that immune responses to infectious pathogens are strictly dependent on the expression of NOS2 [1,5,6]. Arginase is an immune-regulatory enzyme that can reduce the NO production by macrophages, limiting L-arginine availability to NOS2 and inducing resistance of some pathogens to host defense mechanisms [7–10]. In contrast, *Leishmania* also has arginase, which uses L-arginine to produce urea and ornithine as part of polyamine biosynthetic pathway, essential for the parasite replication and establishment of the infection [8,10–15].

The L-arginine uptake in macrophages is mediated by the cationic amino acid transporter family (CAT), such as cationic amino acid transporter 2B (CAT2B) in *Leishmania* infection [16]. By contrast, *Leishmania* has a selective uptake of this amino acid by AAP3 [17,18]. *L*. *amazonensis* AAP3 is encoded by two gene copies (*5*.*1* and *4*.*7 aap3*), arranged *in tandem* on the genome. The open reading frame (ORF) sequence shows 98% identity between the two copies and 93% identity to the AAP3 of *L*. *donovani* (LdAAP3) [18].

Previous studies have demonstrated that *L*. *donovani* responded to amino acid starvation by increasing both mRNA and protein levels of the L-arginine transporter LdAAP3 [17,19]. Castilho-Martins et al. (2011) described an increase in the *5*.*1 aap3* mRNA half-life in *L*. *amazonensis* [18]. *Leishmania* also has mechanisms that sense both external and internal concentrations of L-arginine and can respond with an increase in amino acid uptake [17,18]. Therefore, L-arginine uptake control might be an important factor in the resistance of some pathogens to host defense mechanisms [14,20].

RNA-seq data analyses revealed 14 amino acid transporters differentially expressed in *La*-WT and *La*-arg^-^ promastigotes and axenic amastigotes in the stationary growth phase. The differentiation from promastigotes to axenic amastigotes, independent of arginase activity, showed up-regulation of some these transporters, which may be involved in the *Leishmania* polyamine biosynthetic pathway. In fact, the *5*.*1* and *4*.*7 aap3* transcripts were down-regulated in axenic amastigotes when compared to the promastigote expresson. Up-regulation of other transporters was also identified, as the amino acid transporter aATP11, suggesting that *Leishmania* senses the amino acid pool and regulates gene expression to use alternative route for parasite survival.

In this work, we showed that changes in pH, temperature as well as L-arginine availability and the background from the host macrophage canmodulated the *aap3* mRNA expression and the AAP3 protein amount. We also showed the AAP3 plasma membrane localization correlated with the arginine uptake in *La*-WT mid-logarithmic growth phase promastigotes. The change conditions aimed to simulate the environmental changes of the parasite in its life cycle from the sand fly to the mammalian host. Furthermore, we demonstrated that in addition to its plasma membrane localization, AAP3 was also localized partially in the glycosome of promastigotes and axenic amastigotes forms of the parasite, indicating that arginine uptake can be directed to this compartmentalized organelle, supplementing the polyamine production.

## Methods

### Parasite strain

*L*. *amazonensis* (MHOM/BR/1973/2269) wild type (*La*-WT) and *L*. *amazonensis* arginase knockout (*La*-arg^-^) [8] promastigotes were grown at 25°C in M199 medium supplemented with L-glutamine, 10% heat-inactivated fetal calf serum, 0.25% hemin, 12 mM NaHCO_3_, 100 μM adenine, 40 mM HEPES, 50 U/mL penicillin and 50 μg/mL streptomycin, at pH 7.0. *La*-WT and *La*-arg^-^ axenic amastigotes were grown in M199 medium supplemented, as described above, at 34°C and pH 5.5 [21,22]. *La*-arg^-^ cultures were grown in M199 supplemented as described above with hygromycin (30 μg/mL), puromycin (30 μg/mL) and putrescine (50 μM) addition.

### *In vitro* macrophage infections

For *in vitro* macrophages infection, bone marrow-derived macrophages (BMDMs) from BALB/c or C57BL/6 mice were derived from the femurs and tibiae of females (6-8-weeks) from the Animal Center of the Institute of Bioscience of the University of São Paulo. The femurs and tibiae were washed with cold PBS and the cells were collected at 500 x g for 10 min at 4°C. After lysis of erythrocytes, the cells were maintained in RPMI 1640 medium (LGC Biotecnologia, São Paulo, SP, Brazil), supplemented with penicillin (100 U/ml) (Life Technologies, Carlsbad, CA, USA), streptomycin (100 μg/ml) (Life Technologies, Carlsbad, CA, USA), 5% heat-inactivated FBS (Life Technologies, Carlsbad, CA, USA) and 20% L9-29 supernatant. The cells were cultivated for 7 days at 34°C and 5% CO_2_. After differentiation, cellular viability was evaluated with Trypan blue staining 1:1, and cells were counted in Neubauer chamber. Approximately 2x10^5^ BMDMs were incubated on sterile 8 wells glass chamber slide (Lab-Teck Chamber Slide; Nunc, Naperville, IL, USA), overnight at 34°C and 5% CO_2_ to adhere to the coverslips. Non-adherent cells were removed by PBS washing.

THP-1 human monocytic cell line was maintained in culture at the same conditions for BMDMs. Differentiation was performed plating 5×10^5^ cells in 8 wells chamber slide with 30 ng/mL of phorbol 12-myristate 13-acetate (PMA) (Sigma-Aldrich, St Louis, MO, USA) diluted in RPMI 1640 medium for 72 h, followed by a 72-h resting phase with fresh RPMI 1640 medium before infection.

The infection was performed with *La*-WT or *La*-arg^-^ stationary growth phase promastigotes (MOI 5:1). After 4 h of infection, non-phagocytized parasites were washed with PBS and the cells were collected after 4, 24 and 48 h. Non-infected macrophages maintained in culture in the same conditions were used as control. The infections were evaluated by determining the percentage of infected cells after counting 200 Panoptic-stained (Laborclin, Parana, Brazil) macrophages. The infection index was determined by multiplying the percentage of infected macrophages by the mean number of parasites per infected cell [23,24]. Statistical analyses were performed using non-parametric two-tailed Student *t* tests.

### Total RNA isolation and RT-qPCR

Total RNA of BMDMs from BALB/c or C57BL/6 mice, and THP-1 derived macrophage infected with *La*-WT and *La*-arg^-^ promastigotes; and *La*-WT promastigotes in different conditions of temperature, pH and amino acid starvation or L-arginine supplementation were isolated using TRIzol reagent (Life Technologies, Carlsbad, CA, USA), according to the manufacturer’s instructions. RNA samples were treated with DNase I (Thermo Scientific, Lithuania, EU) and RNA concentration was determined using a spectrophotometer at A260/A280 (Nanodrop ND1000, Thermo Scientific, USA).

For RNA-seq, total RNA from *La*-WT and *La*-arg^-^ promastigotes and axenic amastigotes in the stationary growth phase were isolated using TRIzol reagent (Life Technologies, Carlsbad, CA, USA), according to the manufacturer’s instructions. RNA samples were treated with DNase I (Thermo Scientific, Lithuania, EU). Then, RNA concentration was determined using a spectrophotometer at A260/A280 (Nanodrop ND1000, Thermo Scientific, USA). In addition, RNA integrity was assessed using Agilent 2100 Bioanalyzer and Pico Agilent RNA 6000 kit (Agilent Technologies, Santa Clara, CA, USA), according to the manufacturer’s instructions.

Reverse transcription was performed using 2 μg of total RNA as a template, reverse transcriptase and random primers (Revertaid H minus Reverse Transcriptase kit, Thermo-Scientific, Canada), according to the manufacturer’s instructions. Equal amounts of cDNA were assessed in triplicate in a total volume of 25 μL containing Maxima SYBR Green qPCR Master Mix (Thermo Scientific, Lithuania, EU) and the following primers (200 nM): AAP3_F (5.1 UTR) 5´-GGTCCCCGATACACACATTC-3´, AAP3_R (5.1 UTR) 5´-GTCTCCCGTTTTGCAAGAGA-3´, AAP3_F (4.7 UTR) 5´-ACCATTGTGGGTTAGTTATACATCC-3´, AAP3_R (4.7 UTR) 5´-CAAGATCGC TAGCAGTGGAG-3´, GAPDH_*Leishmania*_F 5´-TCAAGGTCGGTATCAACGGC-3´ and GAPDH_*Leishmania*_R 5´-TGCACCGTGTCGTACTTCAT-3´. The mixture was incubated at 94°C for 5 min, followed by 40 cycles at 94°C for 30 s and 60°C for 30 s. A negative control in the absence of reverse transcriptase was included in RT-qPCR assays to detect DNA contamination in RNA samples. Reactions were carried out using an Exicycler 96 (Bioneer, Daejeon, Korea). The copy number of the target genes (*aap3 5*.*1* and *aap3 4*.*7*) and reference gene (*gapdh*) were quantified in three biological replicate samples, considering the molar mass concentration, according to a standard curve generated from a ten-fold serial dilution of a quantified and linearized plasmid containing the target fragment for each quantification test. The normalized *aap3/gapdh* ratio of the absolute number of molecules of each target was used as the parameter to calculate the relative expression. Analyses were performed using Analysis Exicycler3 Software (Bioneer, Daejeon, Korea).

### Library preparation, RNA-seq and data analysis

cDNA library preparations were performed using Stranded-specific TrueSeq RNA-seq Library Prep (Illumina), according to the manufacturer´s instructions.

Paired-end reads (125 bp) were obtained using the Illumina HiSeq 2000 platform at the Norwegian Sequencing Centre at the University of Oslo. Trimmomatic was used to remove the Illumina adapter sequences [25]. The quality of the produced data was analyzed using FastQC by Phred quality score [26]. Reads with Phred quality scores lower than 20 were discarded. Reads were aligned to the *L*. *mexicana* (MHOMGT2001U1103) genomic data obtained from TriTrypDB (tritrypdb.org/tritrypdb/) using TopHat [27,28]. Thereafter, read mapping was performed for transcript assembly using Cufflinks [29]. After assembly, the abundance of transcripts was calculated as the Fragments Per Kilobase of transcript per Million mapped reads (FPKM), which reflects the abundance of a transcript in the sample by normalization of the RNA length and the total read number [30]. Differentially expressed gene analysis was performed on four comparison pairs (*La*-WT promastigotes vs. *La*-arg^-^ promastigotes; *La*-WT axenic amastigotes vs. *La*-arg^-^ axenic amastigotes, *La*-WT promastigotes vs. *La*-WT axenic amastigotes; *La*-arg^-^ promastigotes vs. *La*-arg^-^ axenic amastigotes) [22].

### Starvation and supplementation assays

Promastigotes in mid-logarithmic growth phase (day 4 of culture) were washed with Earl´s Salt Solution (EBSS) (LGC Biotecnologia, São Paulo, SP, Brazil) at pH 5.0 or pH 7.0. Then, cells were starved of amino acids or supplemented with 400 μM L-arginine for 4 h at 25 or 34°C. The control parasites were those collected before starvation and/or L-arginine supplementation [18].

### Arginine uptake assays

Arginine uptake assays were performed after amino acids starvation or L-arginine supplementation, as previously described [31,32]. Briefly, 5x10^7^ promastigotes in the mid-logarithmic growth phase were washed twice with EBSS medium, resuspended in PBS and incubated at 25°C or 34°C for 3 min. Then, _3_H-arginine (1mCi/43Ci/mmol) (GE Healthcare, UK) was added. The uptake was stopped at different times by adding ice cold arginine. The parasites were washed twice with EBSS and the radioactivity was measured by liquid scintillation spectrometry Perkin-Elmer TRI-CARB 2910TR.

### Production of a rabbit anti-AAP3 polyclonal antibody

The epitope (ILYNFDPVNQP) designed for a specific region of AAP3 through a high affinity MHC was synthesized and used to produce a rabbit anti-AAP3 polyclonal antibody by Proteimax Biotechnology (São Paulo, SP, Brazil).

### Western blot analysis

Approximately 10^7^ parasites in the different conditions were washed with PBS and then lysed with lysis buffer (100 mM Tris-HCl pH 7.5, 2% Nonidet P40, 1 mM PMSF and protease inhibitor cocktail (Sigma-Aldrich, St Louis, MO, USA)). Cells were disrupted by five freeze/thaw cycles in liquid nitrogen and 42°C, and then were cleared of cellular debris by centrifugation at 12,000 x g for 15 minutes at 4°C. Equal amounts of total protein (50 μg) were solved using SDS-PAGE and then transferred to a nitrocellulose membrane (LI-COR Bioscience, Lincoln, NE, USA) using a Trans-Blot Semi-Dry apparatus (Bio-Rad, USA). The membrane was incubated with Blocking Buffer (LI-COR Bioscience, Lincoln, NE, USA) and then with anti-AAP3 serum (1:500 dilution), overnight, at 4°C. After incubation with primary antibody, the membrane was incubated with goat anti-rabbit DyLight 680 conjugated antibody (Thermo Scientific, IL, USA) (1:10000 dilution) for 1 h at room temperature. Anti-α-tubulin (Sigma-Aldrich, St. Louis, MO, USA) (1:5000 dilution) was used to normalize the amount of protein in the blot. All steps were followed by washing 3 times with PBS. The membranes were scanned using an Odyssey CLx apparatus (Li-COR, Lincoln, NE, USA) in 700 channel using an Odyssey System. Odyssey Imaging CLx instrument was used at an intensity setting of 5 (700 nm).

### Flow cytometer analysis

Approximately 10^6^ promastigotes on days 3, 5, 7 and 9 of a growth curve, or in the mid-logarithmic growth phase after amino acid starvation or L-arginine supplementation in pH 7.0 or 5.0 maintained at 25°C or 34°C were washed with PBS and then fixed in 1% of paraformaldehyde (4°C, overnight). For the analysis of AAP3 on the external face of the plasma membrane, the cells were incubated with anti-AAP3 serum (1:500 dilution) at 4°C with overnight shaking. Then, the cells were incubated with goat anti-rabbit FITC conjugated antibody (Sigma-Aldrich, St. Louis, MO, USA) (1:500 dilution) at room temperature with shaking for 1 h. For the analysis of total AAP3, the cells were permeabilized with 0.05% Tween-20 for 20 min at room temperature. Then they were incubated with anti-AAP3 and anti-rabbit FITC, as previously described. The cells were analyzed using FlowSight image flow cytometer (Amnis-MerckMillipore, Darmstadt, Germany). 10,000 cells were acquired, sorted and analyzed using the gate based in gradient root mean square (RMS). Single cells were analyzed using the gate based in the bright field channel and fluorescence intensity of AAP3 in channel 2. Data were acquired using Inspire and analyzed using Ideas Software (Amnis Corporation, Seattle, WA, USA). All analysis was performed at the Core Facility of the Centro de Aquisição de Imagens e Microscopia from Instituto de Biociências (Caimi-IB) at the University of São Paulo.

### Confocal immunolocalization

Approximately 10^6^ promastigotes *La*-WT, *La-*EGFP/SKL [33] or *La*-WT axenic amastigotes in the stationary growth phase were washed with PBS and adhered to coverslips treated with poly-L-lysine (Sigma-Aldrich, St. Louis, MO, USA) for 15 min. The cells were then fixed with 2% paraformaldehyde for 10 min at room temperature. The fixed cells were permeabilized and blocked with 0.1% Triton X-100 and 0.1% BSA in PBS for 1 h at room temperature. To analyze sub-cellular AAP3 localization, anti-AAP3 polyclonal antibody (1:500 dilution) was visualized using an anti-rabbit secondary antibody conjugated to Alexa546 or Alexa 488 (Life Technologies, Carlsbad, CA, USA) (1:500 dilution). Anti-α-tubulin (Life Technologies, Carlsbad, CA, USA) (1:1000 dilution) was visualized using an anti-mouse secondary antibody conjugated to Alexa594 (Life Technologies, Carlsbad, CA, USA) (1:500 dilution). Nuclear and kinetoplast DNA were labeled using DAPI. Each step was followed by washing 10 times with PBS. The coverslips were mounted in ProLong media (Life Technologies, Carlsbad, CA, USA). All imaging was performed using confocal microscope (Zeiss LSM 780 NLO) at the Core Facility of the Centro de Facilidades para Pesquisa (CEFAP) at the University of São Paulo.

### Ethics statement

The experimental protocols for the animals were approved by the Animal Care and Use Committee from the Institute of Bioscience of the University of São Paulo (CEUA 233/2015). This study was carried out in strict accordance with the recommendations in the guide and policies for the care and use of laboratory animals of the São Paulo State (State Law 11.977, de 25/08/2005) and Brazil government (State Law 11.794, de 08/10/2008).

## Results

### Transcriptomic profiling of amino acid transporters of *La*-WT and *La*-arg^-^ promastigotes and axenic amastigotes

Transcriptomic profiling by RNA-seq was used to identify differential gene expression in *La*-WT and *La*-arg^-^ promastigotes and axenic amastigotes. Sequencing data obtained are available on the NCBI BioProject under accession number PRJNA380128 and Sequence Read Archive (SRA) under accession number SRX2661998 and SRX2661999 [22].

More than one billion sequence reads were obtained by Illumina HiSeq2000. Data were aligned to the *L*. *mexicana* genome (MHOMGT2001U1103), and 8253 transcripts, 180 hypothetical proteins and 443 novel transcripts were identified.

Based on the DE genes analyzed, we identified 14 amino acid transporters differentially expressed in the comparisons *La*-WT and *La*-arg^-^ promastigotes and axenic amastigotes. As shown in Tables 1 and 2, we observed a down-regulation of both *5*.*1* and *4*.*7 aap3* in *La*-WT and *La*-arg^-^ promastigotes and axenic amastigotes. In contrast, we observed up-regulation of other amino acid transporters.

**Table 1:** Transcriptomic profiling of amino acid transporters of *La*-WT promastigotes and axenic amastigotes.

ID	*La-WT promastigote vs*. *La-WT axenic amastigote product description*	fold change
LmxM.30.0880	*amino acid permease 3 (aap3 5*.*1)*	0.38
LmxM.30.0870	*amino acid permease 3 (aap3 4*.*7)*	0.44
LmxM.10.0720	*amino acid permease 24*, *putative (AAP24)*	0.71
LmxM.30.0320	*amino acid transporter*, *putative*	0.51
LmxM.16.0210	*mitochondrial ornithine transporter 1-like protein*	1.92
LmxM.27.1580	*amino acid transporter*, *putative*	1.88
LmxM.30.0330	*amino acid transporter aATP11*, *putative*	4.37
LmxM.11.0520	*amino acid permease/transporter*, *putative*	2.22
LmxM.30.0350	*amino acid transporter aATP11*, *putative*	0.57
LmxM.07.1160	*amino acid transporter 19*, *putative (AAT19)*	0.14
LmxM.30.0571	*amino acid transporter aATP11*, *putative*	3.62
LmxM.30.0570	*amino acid transporter aATP11*, *putative*	1.77
LmxM.14.0320	*polyamine transporter 1*, *putative (AAT21)*	1.65
LmxM.16.0210	*mitochondrial ornithine transporter 1-like protein*	1.92

List of down- and up-regulated genes of the total 1,268 previously defined differentially expressed genes in *L*. *amazonensis* wild type (*La*-WT) promastigotes and axenic amastigotes comparisons, adjusted for p < 0.05. The list was based on differentially expressed genes, considering fold change the value obtained from the comparison of the FPKM average for each group plotted. Fold change ≥ 1.5 represented an up-regulation and ≤ 0.5 represented down-regulation.

**Table 2:** Transcriptomic profiling of amino acid transporters of *La*-WT and *La*-arg^-^ promastigotes.

ID	*La-WT promastigote vs*. *La-arg- promastigote product description*	fold change
LmxM.30.0880	*amino acid permease 3 (aap3 5*.*1)*	0.51
LmxM.30.0870	*amino acid permease 3 (aap3 4*.*7)*	0.49
LmxM.30.0350	*amino acid transporter aATP11*, *putative*	2.69

List of down- and up-regulated genes of the total 1,268 previously defined differentially expressed genes in *L*. *amazonensis* wild type (*La*-WT) and *L*. *amazonensis* arginase knockout (*La*-arg^-^) promastigotes, adjusted for p < 0.05. The list was based on differentially expressed genes, considering fold change the value obtained from the comparison of the FPKM average for each group plotted. Fold change ≥ 1.5 represented an up-regulation and ≤ 0.5 represented down-regulation.

Then, to investigate this modulation, we analyzed the changes in environmental signals that could regulate this gene expression, such as pH, temperature and L-arginine availability, as intrinsic factors that can influence the differentiation of the parasite life cycle from the sand fly to the mammalian macrophage host.

### Regulation of *5*.*1* and *4*.*7 aap3* mRNA gene expression in macrophages infected with *La*-WT or *La*-arg^-^

**Fig 1:**
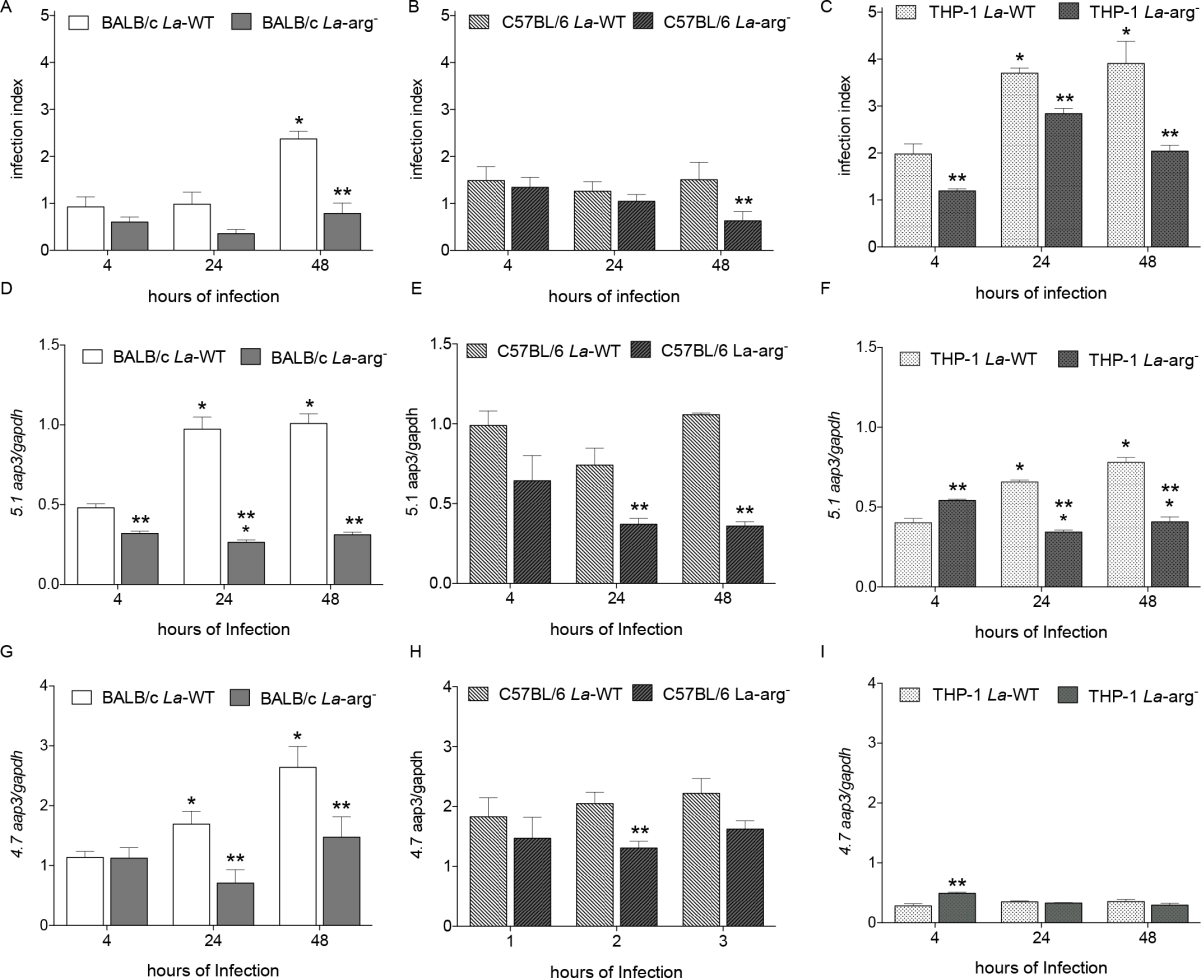
Infection index correlated with *5*.*1* and *4*.*7 aap3* mRNA expression in arginase activity dependent manner. The infection index (A, B and C), the *5*.*1 aap3* copy number (D, E and F) and the *4*.*7 aap3* copy number (G, H and I) were evaluated in BMDMs from BALB/c and C57BL/6 mice, and THP1 derived macrophages, respectively, infected with *La*-WT or *La*-arg^-^ after 4, 24 and 48 h post infection. The transcripts expression was based on the quantification of the target mRNA normalized with *gapdh* expression. Each bar represents the mean ± SEM of the values obtained in 3 independent experiments (n = 4–6). Statistical significance was determined based on non-parametric two-tailed Student *t* tests. (*) p < 0.05, compared to 4 h of infection. (**) p < 0.05, *La*-arg^-^ infected macrophage compared to *La*-WT infected macrophage.

BMDMs from BALB/c or C57BL/6 mice or human lineage THP-1 derived macrophages were infected with *La*-WT or *La*-arg^-^ (MOI 5:1) promastigotes and the infection index was determined at 4, 24 and 48 h post-infection. We did not observe differences in the infection index of BMDMs from BALB/c infected with *La*-WT or *La*-arg^-^ after 4 and 24 h. A lower infection index was observed in BMDMs from BALB/c infected with *La*-arg^-^ after 48 h compared to *La*-WT (Fig 1A), corroborating with previous data and indicating the importance of arginase activity to stablish the infection [8]. The infection index of BMDM from C57BL/6 infected with *La*-WT or *La*-arg^-^ only presented significant difference after 48 h of infection (Fig 1B). In contrast, the infection index from THP-1 macrophages with *La*-WT was increased after 24 and 48 h of infection. And the infection index with *La*-arg^-^ was lower in all time infections when compared to *La*-WT (Fig 1C).

In addition, we determined the *5*.*1* and *4*.*7 aap3* amount in the preparations from macrophages from BALB/c, C57BL/6 and THP-1 infected with *La*-WT or *La*-arg^-^. The *5*.*1 aap3* mRNA amount presented an increase during the time course of macrophages from BALB/c infected with *La*-WT (Fig 1D). The absence of arginase activity did not change the *5*.*1 aap3* mRNA amount during the time course of infection with *La*-arg^-^, but it was lower when compared to *La*-WT infection (Fig 1D). The *5*.*1 aap3* amount in macrophages from C57BL/6 mice infected with *La*-WT also did not change during the time course of infection. (Fig 1E). And in these C57BL/6 macrophages, the absence of arginase activity showed lower expression when compared to *La*-WT infection after 24 and 48 h (Fig 1E). The *5*.*1 aap3* amount in human THP-1 macrophage increased during the time course of infection with *La*-WT. Interestingly, in these THP-1 macrophages, the *5*.*1 aap3* amount was higher in *La*-arg^-^ compared to *La*-WT after 4h of infection, and decreased after 24 and 48 h of infection (Fig 1F). Furthermore, the *4*.*7 aap3* amount appeared up-regulated during the time course of infection in macrophages from BALB/c infected with *La*-WT (Fig 1G). However, did not appear altered in macrophages from BALB/c or C57BL/6 or THP-1 with *La*-arg^-^ infection (Fig 1G, 1H and 1I, respectively). Interestingly, as observed for *5*.*1 aap3*, the *4*.*7 aap3* amount in THP-1 macrophages was higher in *La*-arg^-^ compared to *La*-WT after 4h of infection (Fig 1I).

### Regulation of *5*.*1* and *4*.*7 aap3* mRNA gene expression in *La*-WT promastigotes in changes of pH, temperature and availability of L-arginine

**Fig 2:**
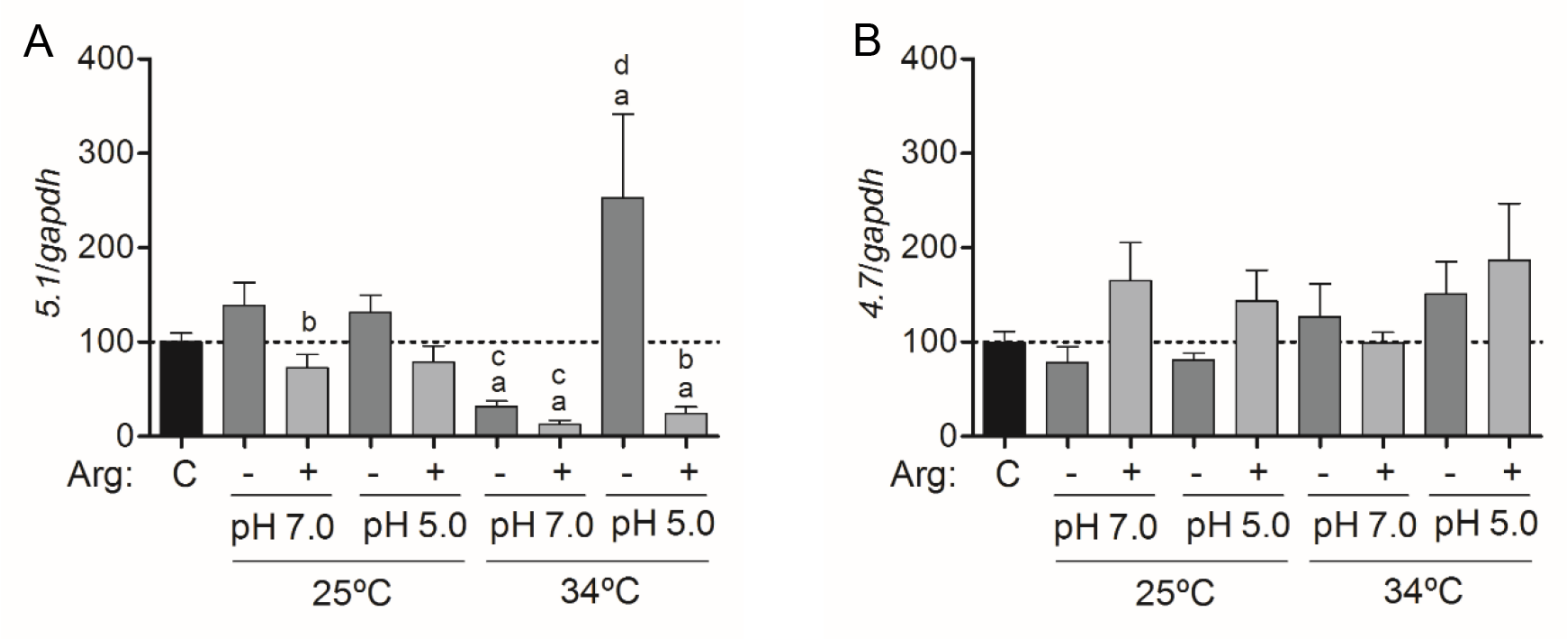
Shift in pH, temperature and L-arginine availability influences *5*.*1 aap3* transcripts amount. The *5*.*1* and *4*.*7 aap3* mRNA levels were based on quantification of the target and were normalized by *gapdh* expression. **(A)** Copy number of *5*.*1 aap3* transcripts determined after starvation or supplementation with 400 μM L-arginine at 25 and 34°C, and in pH 5.0 or pH 7.0. (a) p < 0.05, comparing the control and the changed conditions of starvation (-) or L-arginine supplementation (+) at 25°C or 34°C and in pH 5.0 or pH 7.0. (b) p < 0.05, comparing the changed conditions of starvation or L-arginine supplementation. (c) p < 0.05, comparing the changed conditions at 25°C or 34°C in pH 5.0 and pH 7.0. (d) p < 0.05, comparing the changed conditions at 25°C and 34°C in pH 5.0 or pH 7.0. **(B)** Copy number of *4*.*7 aap3* transcripts determined after starvation or supplementation with 400 μM L-arginine at 25 and 34°C, and in pH 5.0 and pH 7.0. The values are the mean ± SEM of three independent biological replicates. (C) Control parasites were collected before amino acid starvation and/or L-arginine supplementation.

*La*-WT promastigotes in mid-logarithmic growth phase were starved of amino acids or supplemented with 400 μM L-arginine and incubated at 25°C or 34°C for 4 h. Then, total RNA was extracted and the *5*.*1* and *4*.*7 aap3* mRNA were quantified by RT-qPCR. Data were normalized to the *gapdh* transcripts in each condition. As shown in Fig 2A, in parasites maintained at 25°C and pH 7.0, the starvation of L-arginine caused a significant increase in *5*.*1 aap3* transcript level compared with parasites that were supplemented with L-arginine. Interestingly, at 34°C, an increase in *5*.*1 aap3* transcripts was observed during starvation of L-arginine at pH 5.0 (Fig 2A). By contrast, a significant decrease of the same transcript was observed at pH 7.0 during starvation as well asin parasites submitted to L-arginine supplementation. The decrease was also detected at pH 5.0 during L-arginine supplementation (Fig 2A).

Although no significant difference was observed in the amount of *4*.*7 aap3* transcripts in all conditions, a slight decrease in the mRNA was observed when the parasites were submitted to amino acid starvation at 25°C (Fig 2B). The profiles at 25°C and 34°C were very similar, except at 34°C in pH 5.0, when starvation slightly increased the mRNA level (Fig 2B). Our data suggest that L-arginine availability and increased temperature regulates only *5*.*1 aap3* mRNA expression.

### AAP3 protein expression in *La*-WT promastigotes in changes of pH, temperature and L-arginine availability

To assess the amount of AAP3 present on the external face of the plasma membrane and the total AAP3 present in the whole cell, we performed a flow cytometry analysis of fluorescence labeled AAP3 antibody against non-permeabilized and permeabilized parasites. The fluorescence intensity values were normalized to the control (parasite collected before amino acid starvation or L-arginine supplementation). Initially, the total amount of AAP3 protein was measured during the time course of the growth curve. We observed an increase of AAP3 protein on days 3, 5, 7 and 9 compared to day 2 (S1 Fig). This data indicated that the increase of AAP3 in the plasma membrane was related from mid-logarithmic to late-stationary growth phase. Then, the total amount of AAP3 was measured during pH and temperature changes, and L-arginine availability.

**Fig 3:**
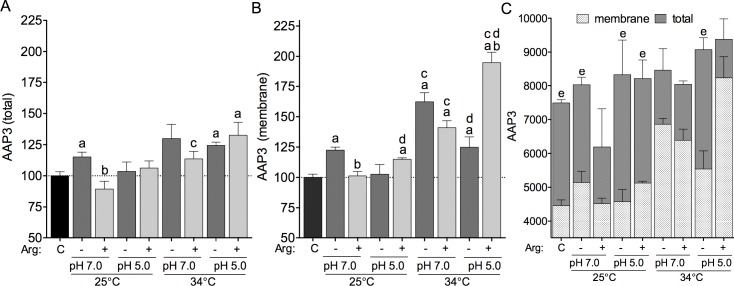
Shift in pH, temperature and L-arginine availability influences AAP3 protein expression and intracellular organization. AAP3 protein expression was determined after starvation or supplementation with 400 μM L-arginine at 25 and 34°C, and in pH 5.0 and pH 7.0. **(A)** Fluorescence detection of AAP3 protein on the permeabilized parasites. **(B)** Fluorescence detection of AAP3 protein on the plasma membrane of non-permeabilized parasites. **(C)** Absolute quantification of cells and comparison of plasma membrane and total AAP3 protein. (a) p < 0.05, comparing the control and the changed conditions of starvation (-) or L-arginine supplementation (+) at 25°C or 34°C and in pH 5.0 or pH 7.0. (b) p < 0.05, comparing the changed conditions of starvation or L-arginine supplementation. (c) p < 0.05, comparing the changed conditions at 25°C or 34°C in pH 5.0 and pH 7.0. (d) p < 0.05, comparing the changed conditions at 25°C and 34°C in pH 5.0 or pH 7.0. (e) p < 0.05, comparing the membrane AAP3 to total AAP3. The values are the mean ± SEM of 4–6 independent biological replicates. (C) Control parasites were collected before amino acid starvation and/or L-arginine supplementation.

The total amount of AAP3 protein was increased in parasites kept at 25°C and pH 7.0 under amino acid starvation, compared to the control parasites, but not in the parasites at pH 5.0 (Fig 3A). Interestingly, a decreased of total amount was observed in parasites at 25°C, pH 7.0 and supplemented with L-arginine when compared to parasites under amino acids starvation. No difference in the total AAP3 amount was observed in parasites at 34°C and pH 7.0, compared to the control parasites. In contrast, an increase in the total amount was observed in parasites at pH 5.0 independent of amino acids starvation or L-arginine supplementation (Fig 3A). Furthermore, the plasma membrane amount of AAP3 was measured and we observed that the parasites under amino acid starvation at 25°C in pH 7.0 presented increased AAP3 amount in the membrane compared to the control parasites (Fig 3B). A decreased in the membrane amount was observed in parasites at 25°C, pH 7.0, supplemented with L-arginine when compared to parasites under amino acids starvation. No significant difference was observed at pH 5.0 under amino acids starvation, compared to control parasites. However, an increased membrane AAP3 amount was observed at pH 5.0 under L-arginine supplementation when compared to the control parasites and to those at pH 7.0. The AAP3 membrane amount was increased at 34°C, in both pH 7.0 and pH 5.0, and in amino acid starvation or supplementation of L-arginine (Fig 3B). The differences in the total and plasma membrane AAP3 amount reflected the increase in the protein expression and in the traffic of the transporter carrier to the membrane. The increase in total and plasmatic membrane AAP3 amount occurred in amino acid starvation at 25°C and pH 7.0, and at 34°C and pH 5.0, contrasting with the amount of total AAP3 and plasmatic membrane in L-arginine supplementation at 34°C (Fig 3C).

In addition, we performed Western blot analysis of cell lysates of *La*-WT promastigotes and axenic amastigotes during the time course of growth curve, and no difference was observed in the AAP3 protein level (S2A Fig). Compared with the flow cytometry analysis results of total AAP3 protein (Fig 3C), the protein level detected by Western blotting showed a similar profile in *La*-WT promastigotes after amino acid starvation or L-arginine supplementation at both 25°C and 34°C (S2C and S2D Fig).

### Arginine transport in *La*-WT promastigotes in changes of pH, temperature and L-arginine availability

To evaluate and correlate the results obtained for mRNA and protein levels, we analyzed L-arginine uptake in parasites submitted to the same pH and temperature changes, and L-arginine availability.

**Fig 4:**
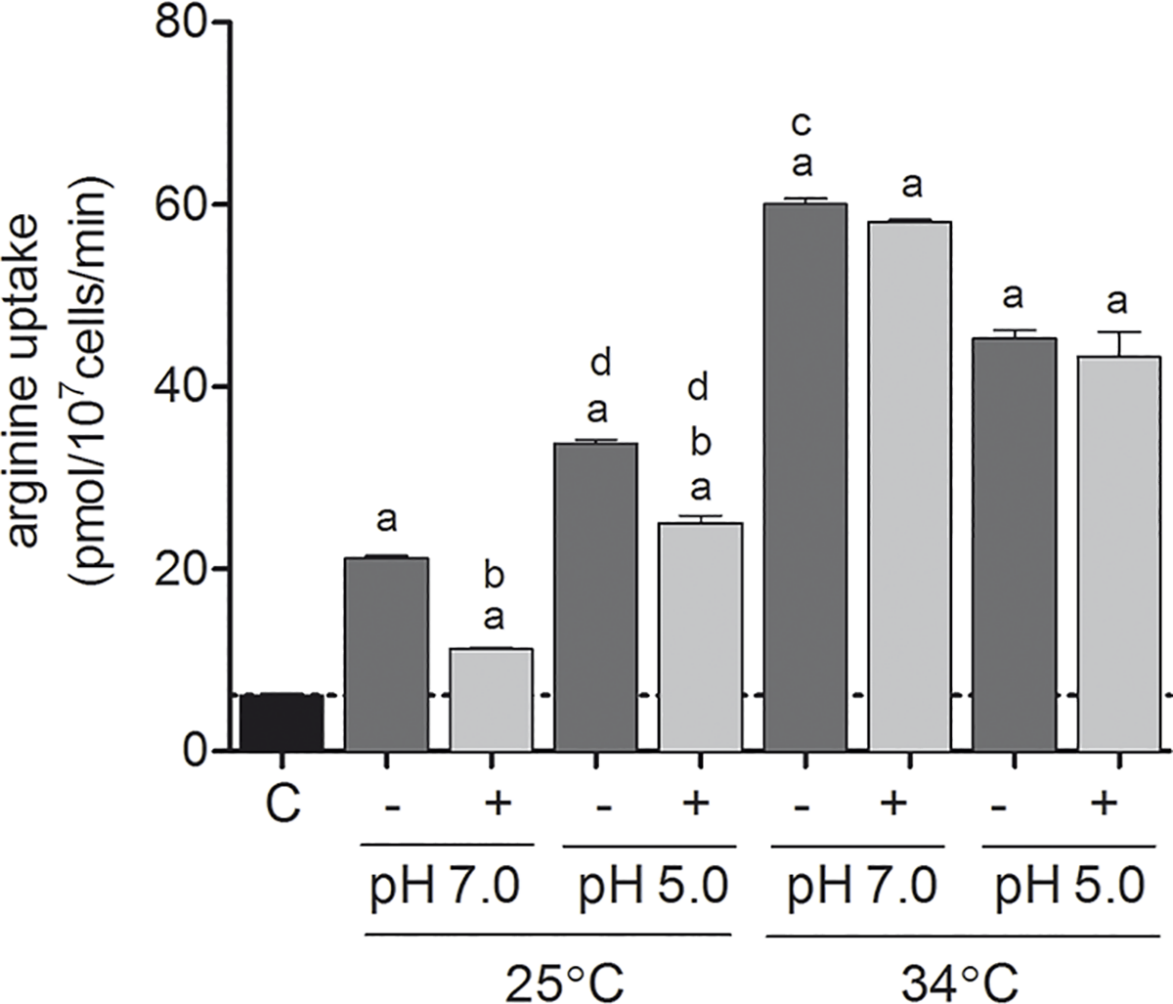
Shift in pH, temperature and L-arginine availability influences L-arginine uptake. H^3^L-arginine uptake was available after starvation or supplementation with 400 μM L-arginine at 25 and 34°C, and in pH 5.0 and pH 7.0. The transport was stopped with excess L-arginine, on ice, and washed 3-times with PBS. The cells were resuspended in scintillation liquid and scintillation count values were converted to pmol/min and normalized per number of parasites (1x10^7^). (a) p < 0.05, comparing the control and the changed conditions of starvation (-) or L-arginine supplementation (+) at 25°C or 34°C and in pH 5.0 or pH 7.0. (b) p < 0.05, comparing the changed conditions of starvation or L-arginine supplementation. (c) p < 0.05, comparing the changed conditions at 25°C or 34°C in pH 5.0 and pH 7.0. (d) p < 0.05, comparing the changed conditions at 25°C and 34°C in pH 5.0 or pH 7.0. The values are the mean ± SEM of three independent biological replicates.

The L-arginine uptake increased during the amino acid starvation at pH 7.0 (Fig 4), demonstrating a correlation with the increase exhibited by *5*.*1 aap3* mRNA in the same conditions (Fig 2A). Changing the parasites to 34°C caused a 3-times increase in the rate of L-arginine uptake independent of pH, as well as amino acid starvation or L-arginine supplementation (Fig 4).

### Immunolocalization of AAP3 in *L*. *amazonensis* promastigotes and axenic amastigotes

**Fig 5:**
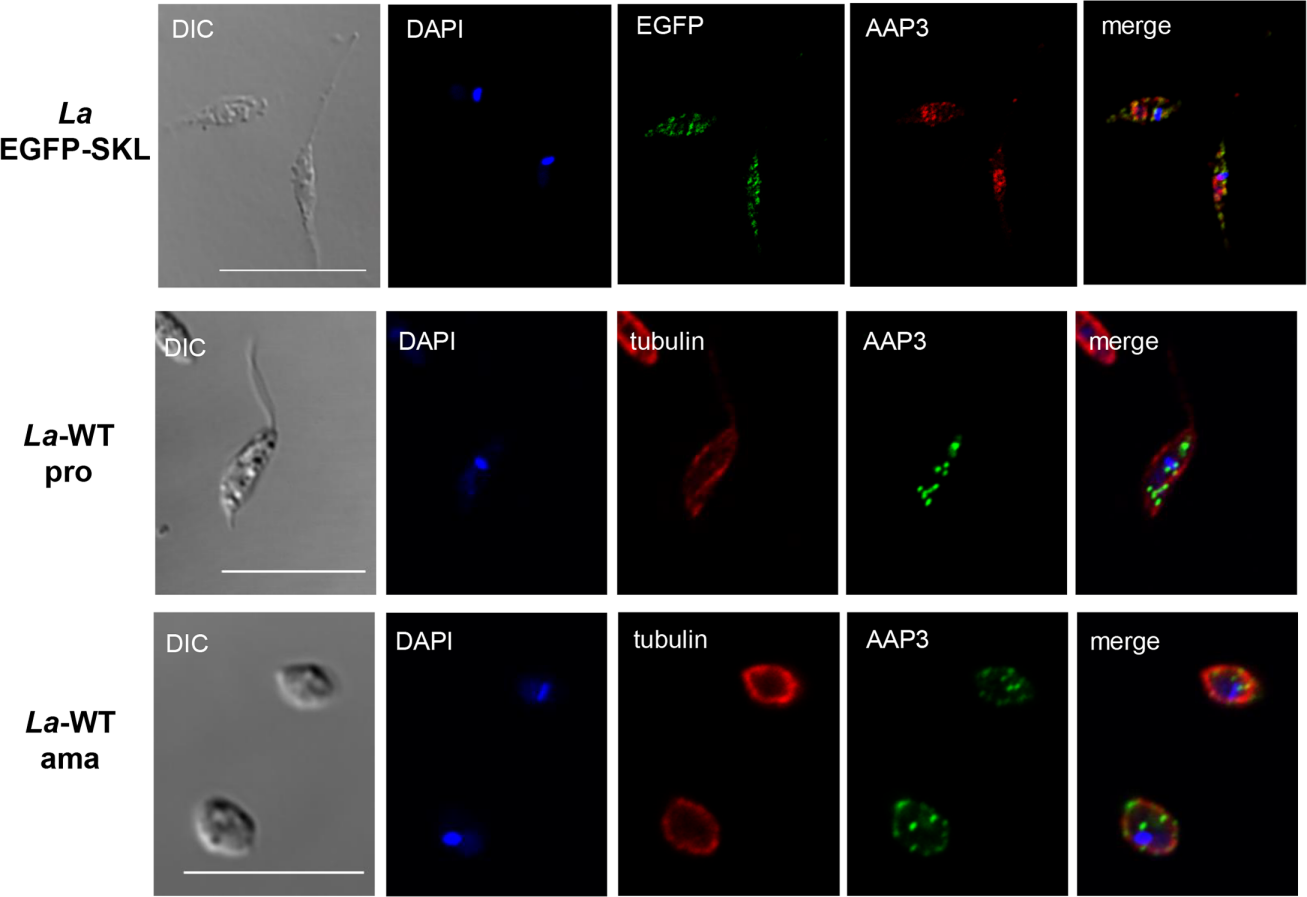
Cellular localization of AAP3 in the plasma membrane and glycosome in both promastigotes and axenic amastigotes. EGFP-SKL (*La*-EGFP-SKL) promastigotes in the stationary growth phase: DIC image; DNA stained using DAPI (blue); EGFP/SKL (green); anti-AAP3 polyclonal antibody visualized with an anti-rabbit secondary antibody conjugated to Alexa546 (red); and merged image. *L*. *amazonensis* (*La*-WT) promastigotes (pro) and axenic amastigotes (ama) in the stationary growth phase: DIC image; DNA stained using DAPI (blue); anti-AAP3 polyclonal antibody visualized with an anti-rabbit secondary antibody conjugated to Alexa488 (green); anti-α-tubulin visualized with an anti-mouse secondary antibody conjugated to Alexa594 (red); and merged image. All images were acquired using a Zeiss LSM confocal microscope. Bar 10μm.

Using the anti-rabbit antibody against the epitope AAP3, confocal microscopy analysis was performed to localized that transporter in the parasites. Confocal images from *La*-WT promastigotes and axenic amastigotes in the stationary growth phase, and *La*-GPF/SKL promastigotes in the stationary growth phase confirmed the AAP3 localization in the plasmatic membrane as well as partially in the glycosome (Fig 5).

## Discussion

The importance of the amino acid L-arginine in *Leishmania* has been related to parasite replication as well as a requirement to establish the infection in the mammalian host [8,17,32,34][22,35]. In the course of the *Leishmania* life cycle, environmental changes may act as signals to regulate gene expression, which enables the parasite to adapt to the new conditions [7].

Stress signaling starts with the consumption of all available nutrients at the end of blood meal digestion in the insect's digestive tract. The starvation signal can cause the release of procyclic promastigotes from the insect mid-gut epithelia and its migration to the proboscis, promoting the differentiation of procyclic promastigotes into metacyclic promastigotes [36]. The deprivation of amino acids is an important signal for metacyclogenesis, providing the parasites with the infective capacity to establish the infection.

The shift of temperature, from the sand fly (25^°^C) to the mammalian body temperature (37°C), is the next shock and is also known to be an important signal for parasite differentiation. The heat-shock proteins are good examples of gene activation that allow parasite survival in rapid temperature changes [37–39]. The pH change is the following step upon the fusion of the phagosome to the lysosome in the formation of the phagolysosome [37,40,41].

In this context, we described here how the parasite was able to regulate the AAP3 expression in response to the L-arginine availability, arginase activity, pH and temperature modifications. AAP3 is an amino acid transporter described for L-arginine uptake in *L*. *amazonensis* and *L*. *donovani* [17,18]. This transporter is encoded by two copies of *aap3* gene (*5*.*1* and *4*.*7*). A possible explanation about the presence of these two copies can be related with the post-transcriptional gene regulation according to the environmental conditions, since the two open reading frames are similar [18].

The differentially gene expression based on the RNA-seq analysis in *La*-WT and *La*-arg^-^ promastigotes and axenic amastigotes in the stationary growth phase revealed transcripts of amino acid transporters down- and up-regulated. The *5*.*1* and *4*.*7 aap3* appeared down-regulated in the comparisons of *La*-WT promastigotes vs. axenic amastigotes and *La*-WT vs. *La*-arg^-^ promastigotes. Similar results were observed in RT-qPCR assays from intracellular *La*-WT or *La*-arg^-^ amastigotes infecting macrophages with different genetic background (BALB/c and C57BL/6 mice, and human THP-1). Interestingly, in THP-1 macrophages, we observed an increased expression of both *5*.*1* and *4*.*7 aap3* after 4 h of infection with *La*-arg^-^ when compared to *La*-WT. This differential behavior can be explained based on the distinct genetic background of the host that can influence in L-arginine accumulation. Then, in THP-1 and *La*-arg^-^ infections, the absence of arginase activity can lead to a host transporter modulation. By its side, the parasite can respond to the L-arginine availability modulating the *aap3* expression to establish the infection.

Previous studies demonstrated increased *aap3* mRNA expression during the time course of macrophage infection. Muxel et al., 2017 showed that intracellular amastigotes increased the amount of *5*.*1 aap3* in the time course of macrophage infection with *La*-WT. On the other hand, the infection with *La*-arg^-^ did not altered *5*.*1 aap3* levels. These data demonstrated the importance of L-arginine availability and arginase activity to regulate *5*.*1 aap3* mRNA expression and L-arginine transport to ensure the amastigote survival [7]. Additionally, when the L-arginine availability was lower in melatonin-treated infected macrophages, the levels of *5*.*1 aap3* and arginase mRNA of intracellular amastigotes were maintained on trial to keep the polyamine supply [42]. Goldman-Pikovich et al., 2016 showed that *L*. *donovani* intracellular amastigotes presented higher levels of *LdAAP3*.*2* mRNA than in axenic amastigotes kept in L-arginine-starvation condition [19]

According to TriTryp database, we identified AAP3 orthologs in *L*. *major* (LMJLV39_310014600, LMJLV39_310014700, LMJSD75_310014300, LMJSD75_310014400, LmjF.31.0870 and LmjF.31.0880), *L*. *gerbilli* (LGELEM452_310014200), *L*. *tropica* (LTRL590_310015200 and LTRL590_310015300), *L*. *turanica* (LTULEM423_310014200), *L*. *braziliensis* (LbrM.31.1030; LBRM2903_000006400 and LBRM2903_310017700), *L*. *donovani* (LdBPK_310900.1 and LdBPK_310910.1) and *L*. *infantum* (LinJ.31.0900 and LinJ.31.0910). The AAP3 transcripts were annotated for *L*. *donovani* (LdBPK_310900.1.1) [43], *L*. *infantum* (LinJ.31.0910) [31], *L*. *major* (LmjF.31.0870) [44] and *L*. *mexicana* (LmxM.30.0870.1) [45] (TriTrypDB). These findings among the different *Leishmania* spp. show the importance of L-arginine metabolism and uptake indicating that it could contribute to the fine tuning of gene expression and consequently L-arginine uptake.

Still, according to the RNA-seq data, we also identified other amino acid transporters differentially expressed in the following comparisons: *La*-WT promastigotes vs. axenic amastigotes, and *La*-WT vs. *La*-arg^-^ promastigotes. The transcript aATP11, an amino acid transporter, was previously described in association with amino acid starvation [19], confirmed the gene expression regulation in stress conditions. Notably, some aATP11 members were up-regulated (LmxM.30.0330, LmxM.30.0571 and LmxM.30.0570) and other was down-regulated (LmxM.30.0350). Other studies have shown that amastigotes activate signals when internalized into the phagolysosome, which has been linked to the down-regulation of many surface nutrient transporters. The remodeling of amastigote central carbon metabolism also represents a programmed response to stress that cells undergo in the host macrophage [46,47].

The response to starvation or L-arginine supplementation in different conditions of pH and temperature revealed that *5*.*1 aap3* mRNA was down-regulated at 34°C and pH 7.0 in both amino acid starvation and L-arginine supplementation, and at pH 5.0 in L-arginine supplementation. These observations can indicate a gene expression regulation at 34°C, during amastigote differentiation. On the other hand, *5*.*1 aap3* mRNA was up-regulated at pH 5.0 in amino acid starvation, as previously described for *L*. *amazonensis* [18] and *L*. *donovani* [19], suggesting up-regulation of the transporter in response to low L-arginine availability. The *4*.*7 aap3* mRNA did not show significant expression differences, indicating that probably only the *5*.*1 aap3* copy present the regulatory sequence for modulation.

The data obtained after L-arginine starvation showed increased amino acid uptake corroborating previous findings [18], indicating that *Leishmania* senses the concentration of this amino acid and regulates the expression of the transporter [17–19]. L-arginine starvation reduced the levels of arginine, ornithine and putrescine, but not spermidine, spermine and agmatine, in *L*. *amazonensis* promastigotes [48]. This condition allows the parasite to sense new signals, such as L-arginine availability in the phagolysosome environment, which is predominantly caused by the competition for L-arginine with the host cell [19]. The absence of L-arginine or polyamine could be surpassed by the polyamine transporter, described and characterized in *L*. *major*, with high affinity for putrescine and spermidine [49], *L*. *donovani* [50,51] and *L*. *mexicana* [50], and presenting an optimal transport function at pH 7.0–7.5 for promastigotes and pH 5.0 for amastigotes of *L*. *mexicana* [50]

The increase of AAP3 protein levels in the plasmatic membrane reflected the increase of L-arginine uptake at 34°C, highlighting the participation of AAP3 and the importance of the uptake of the amino acid during promastigote to amastigote differentiation, as a response to the change in temperature (25 to 34°C) [17–19]. And the increase amount of total AAP3 compared to the plasma membrane can suggest the directing of this transporter to other compartments. This hypothesis was confirmed with the cellular localization of AAP3 in promastigotes and axenic amastigotes. Previous studies already demonstrated the LdAAP3 localized in the flagella surface and in the glycosome [19]. As arginase enzyme was also localized in the glycosome [8] and as Szoor et al. (2010) reported that signaling in the nutrient-sensing pathway was targeted to this organelle in *Trypanosoma brucei* [52], we hypothesized that the AAP3 could also be localized in the glycosome to supply polyamines biosynthesis. In this study, we demonstrated AAP3 localized in the plasma membrane as well as in the glycosome of *La*-WT promastigote and axenic amastigotes in the stationary growth phase, indicating that L-arginine uptake is directed to this organelle. The results presented in this communication indicated that as a strategy for controlling *Leishmania* infection could be focused in the inhibition of L-arginine flux into the glycosome of the parasite.

## Supporting information

S1 Fig AAP3 protein growth curve of *L. amazonensis* promastigotes based on flow cytometry. The quantification of fluorescence intensity of anti-AAP3 was performed by image flow cytometry of permeabilized (total protein–gray) or non-permeabilized (plasma membrane protein–light gray) parasites. The values are the mean ± SEM of 3 independent biological replicates. (e) p < 0.05, comparing the membrane to total AAP3.
(TIFF)

S2 Fig AAP3 protein expression in *La*-WT, determined by Western blotting. Total extracts during the time-course of *La*-WT promastigotes and axenic amastigotes in the stationary phase, and *La*-WT promastigotes after starvation and supplementation with 400 μM L-arginine. Promastigotes were lysed and the proteins were separated by SDS-PAGE, transferred to nitrocellulose membrane and immunoblotted with an anti-AAP3 polyclonal antibody. An anti-α-tubulin antibody was used as a control. The images were scanned using an Odyssey CLx imaging system (Li-COR). **(A)** AAP3 protein expression levels during the time course of the *La*-WT promastigotes growth curve. **(B)** AAP3 protein expression levels during the time course of the *La*-WT axenic amastigotes growth curve **(C)** AAP3 protein expression in promastigotes in stationary growth phase after starvation (-) or L-arginine supplementation (+) at 25°C in pH 7.0 or 5.0. **(D)** AAP3 protein expression in stationary phase promastigotes after starvation and supplementation with 400 µM L-arginine at 34°C in pH 7.0 and 5.0. *La*-WT *L*. *amazonensis* wild type. (C) Control parasites were collected before amino acid starvation and/or L-arginine supplementation.
(TIF)
