# Supplementary figures and images for: L-arginine availability and arginase activity: Characterization of amino acid permease 3 in Leishmania amazonensis
Source: PLoS Negl Trop Dis. 2017 Oct 26;11(10):e0006025. doi: 10.1371/journal.pntd.0006025 (PMC5693463; doi:10.1371/journal.pntd.0006025)

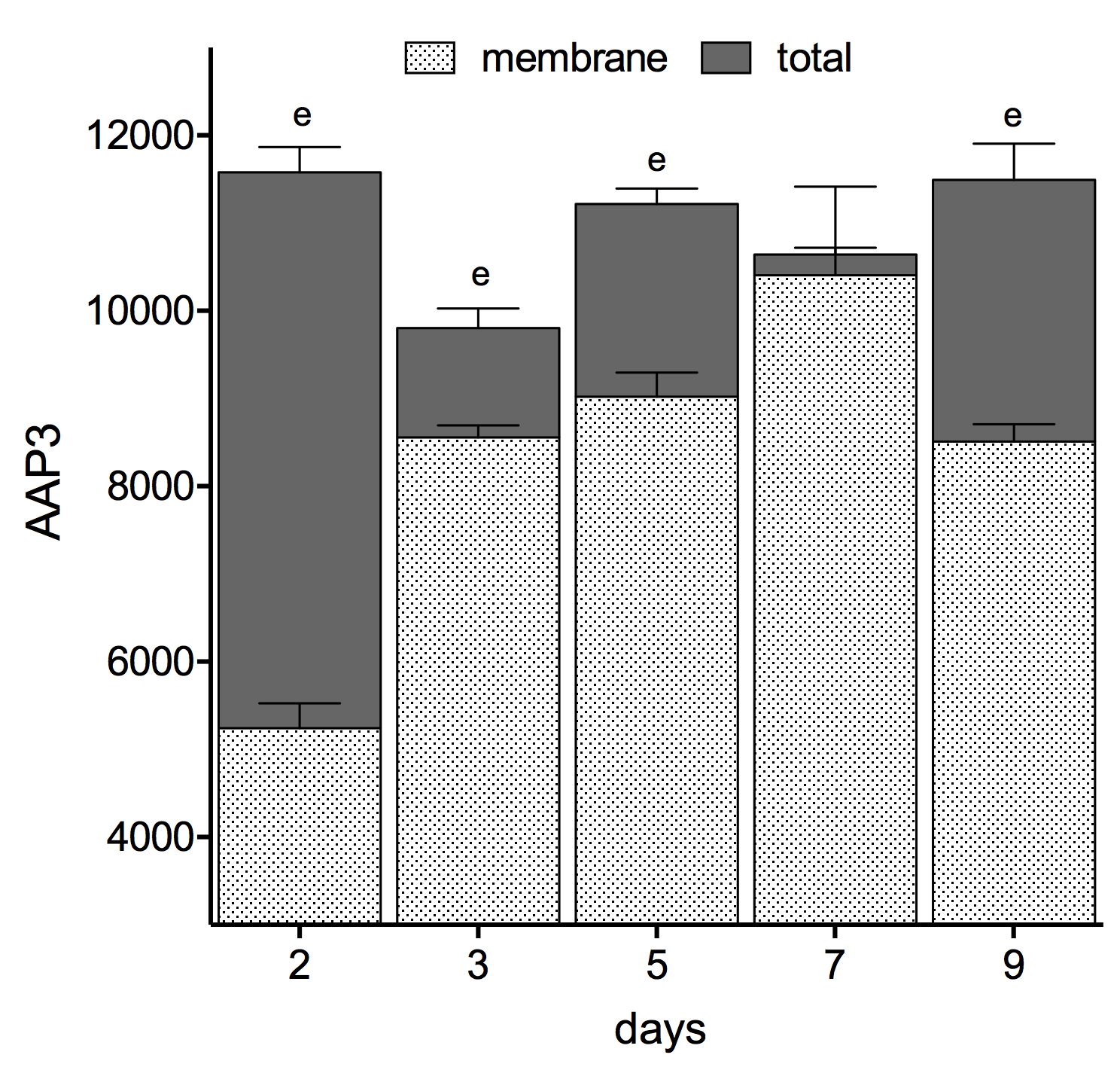

Supplement: S1 Fig — The quantification of fluorescence intensity of anti-AAP3 was performed by image flow cytometry of permeabilized (total protein–gray) or non-permeabilized (plasma membrane protein–light gray) parasites. The values are the mean ± SEM of 3 independent biological replicates. (e) p < 0.05, comparing the membrane to total AAP3. (TIFF) [file pntd.0006025.s001.tiff]

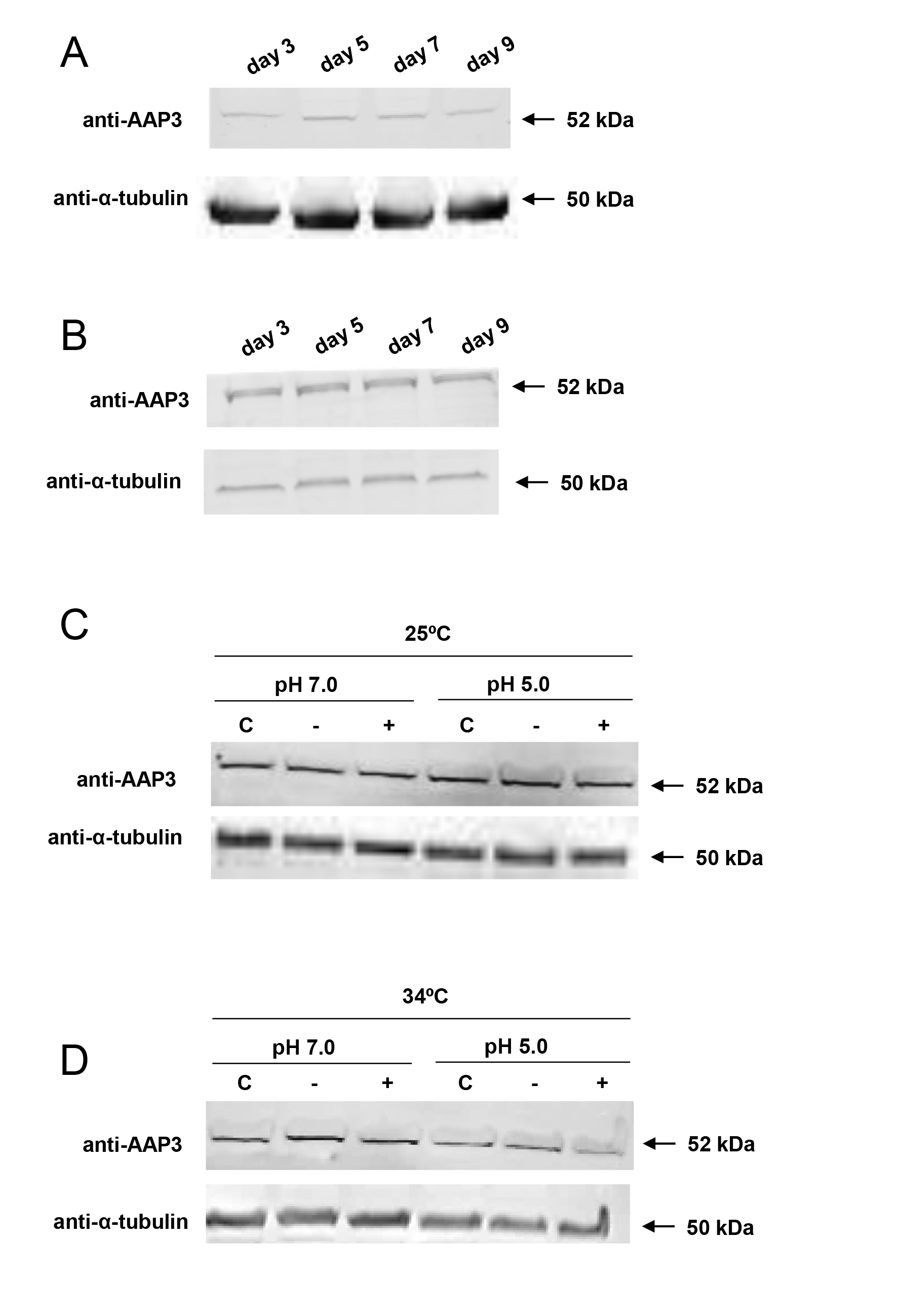

Supplement: S2 Fig — Total extracts during the time-course of La-WT promastigotes and axenic amastigotes in the stationary phase, and La-WT promastigotes after starvation and supplementation with 400 μM L-arginine. Promastigotes were lysed and the proteins were separated by SDS-PAGE, transferred to nitrocellulose membrane and immunoblotted with an anti-AAP3 polyclonal antibody. An anti-α-tubulin antibody was used as a control. The images were scanned using an Odyssey CLx imaging system (Li-COR). (A) AAP3 protein expression levels during the time course of the La-WT promastigotes growth curve. (B) AAP3 protein expression levels during the time course of the La-WT axenic amastigotes growth curve (C) AAP3 protein expression in promastigotes in stationary growth phase after starvation (-) or L-arginine supplementation (+) at 25°C in pH 7.0 or 5.0. (D) AAP3 protein expression in stationary phase promastigotes after starvation and supplementation with 400 µM L-arginine at 34°C in pH 7.0 and 5.0. La-WT L. amazonensis wild type. (C) Control parasites were collected before amino acid starvation and/or L-arginine supplementation. (TIF) [file pntd.0006025.s002.tif]
